# Nursing students experience during the COVID-19 pandemic: a qualitative research** ***

**DOI:** 10.17533/udea.iee.v40n2e13

**Published:** 2022-09-20

**Authors:** Ximena Farfán-Zúñiga, Paula Jaman-Mewes, Melissa Zimmermann-Vildoso, Claudia Campos-Lobos

**Affiliations:** 1 Nurse, M.Sc. Professor. Facultad de Enfermería y Obstetricia, Universidad de los Andes, Chile. Email: xfarfan@uandes.cl Facultad de Enfermería y Obstetricia Universidad de los Andes Chile xfarfan@uandes.cl; 2 Nurse, M.Sc, Ph.D candidate. Professor. Facultad de Enfermería y Obstetricia, Universidad de los Andes, Chile. Email: pjaman@uandes.cl Facultad de Enfermería y Obstetricia Universidad de los Andes Chile pjaman@uandes.cl; 3 Nurse, M.Sc, Ph.D candidate. Professor. Facultad de Enfermería y Obstetricia, Universidad de los Andes, Chile. Email: mzimmermann@uandes.cl Facultad de Enfermería y Obstetricia Universidad de los Andes Chile mzimmermann@uandes.cl; 4 Nurse, M.Sc. Professor. Facultad de Enfermería y Obstetricia, Universidad de los Andes, Chile. Email: ccampos@uandes.cl Facultad de Enfermería y Obstetricia Universidad de los Andes Chile ccampos@uandes.cl

**Keywords:** COVID-19, pandemics, students, nursing, nursing care, qualitative research, COVID-19, pandemias, estudiantes de enfermería, atención de enfermería, investigación cualitativa., COVID-19, pandemias, estudantes de enfermagem, cuidados de enfermagem, pesquisa qualitativa

## Abstract

**Objective.:**

To describe the professional practice experiences of fifth year nursing students during the COVID-19 pandemic.

**Methods.:**

Qualitative research design with content analysis. Participant sampling was purposive. 13 fifth-year nursing students participated. All of them completed their professional clinical practice in public hospitals and private clinics who cared for COVID-19 patients in Chile. The data were obtained through guided online written self-reflections.

**Results.:**

Three main themes were inductively identified: (1) Facing with a very difficult and stressful situation, due to the permanent use of personal protection elements, multiple emotions, and physical fatigue, as well as facing ethical-clinical dilemmas in daily tasks; (2) Recognising different coping styles in difficult moments, highlighting contact with significant people and combination form of support and harmful ways of coping with stress; and (3) Experiences disciplinary learning and personal growth, such as: nursing care management, interpersonal skills in times of crisis and having contributed to the country in this adverse context.

**Conclusion.:**

The clinical experience of the students in times of COVID-19, was an opportunity to learn how to perform nursing care in times of crisis, humanize care and support health teams in some of the country's hospitals.

## Resumen

**Objetivo.:**

Describir las experiencias de la práctica profesional de los estudiantes de quinto año de enfermería durante la pandemia de COVID-19.

**Métodos.:**

Diseño de investigación cualitativa con análisis de contenido. El muestreo de participantes fue intencional. Participaron 13 estudiantes de quinto año de enfermería. Todos ellos realizaron su práctica clínica profesional en hospitales públicos y clínicas privadas atendiendo a pacientes con COVID-19 en Chile.

**Resultados.:**

Se identificaron inductivamente tres temas principales: (1) Enfrentarse a una situación muy difícil y estresante, debido al uso permanente de elementos de protección personal, a las múltiples emociones y al cansancio físico, así como a dilemas ético-clínicos en las tareas diarias; (2) Reconocimiento de los diferentes estilos de afrontamiento en momentos difíciles, destacando el contacto con personas significativas, y la combinación de formas de apoyo y formas perjudiciales de afrontar el estrés; y (3) Experiencias de aprendizajes disciplinares y de crecimiento personal, tales como: gestión de cuidados de enfermería, habilidades interpersonales en momentos de crisis y haber contribuido al país en este contexto adverso.

**Conclusión.:**

La experiencia clínica de los estudiantes en tiempos del COVID-19 fue una oportunidad para aprender a realizar cuidados de enfermería en tiempos de crisis, humanizar la atención y apoyar a los equipos de salud en algunos hospitales del país.

## Resumo

**Objetivo.:**

Descrever as experiências de prática profissional de estudantes de enfermagem do quinto ano durante a pandemia de COVID-19.

**Métodos.:**

Desenho de pesquisa qualitativa com análise de conteúdo. A amostragem dos participantes foi intencional. Participaram 13 estudantes de enfermagem do quinto ano. Todos eles realizaram seu estágio clínico profissional em hospitais públicos e clínicas privadas atendendo pacientes com COVID-19 no Chile.

**Resultados.:**

Três temas principais foram identificados indutivamente: (1) Enfrentar uma situação muito difícil e estressante, devido ao uso permanente de elementos de proteção individual, emoções múltiplas e cansaço físico, bem como enfrentar dilemas ético-clínicos nas tarefas diárias; (2) Reconhecimento dos diferentes estilos de enfrentamento em momentos difíceis, destacando o contato com pessoas significativas, e a combinação de formas solidárias e prejudiciais de enfrentamento do estresse; e (3) Experiências de aprendizagem disciplinar e crescimento pessoal, tais como: gestão do cuidado de enfermagem, habilidades interpessoais em tempos de crise e ter contribuído para o país neste contexto adverso.

**Conclusão.:**

A experiência clínica dos alunos em tempos de COVID-19 foi uma oportunidade para aprender a realizar a assistência de enfermagem em tempos de crise, humanizar o atendimento e apoiar as equipes de saúde em alguns hospitais do país.

## Introduction

A new global virus called SARS-CoV-2 emerged in the Chinese city of Wuhan in December 2019, it was called COVID-19 by the World Health Organisation.^1^ It has caused a great impact in the lives of people, causing more than 3 million deaths globally and has impacted every country. Chile has not been the exception with ominous effects on the economy, politics and education, and at personal and family level of every citizen.^2^ In March 2020, the WHO announced a pandemic for COVID-19. In Chile, a catastrophic constitutional state of exception was decreed, to contain and overcome the health crisis.^3^ The transmission and severity of the disease has caused the death of more than 35,000 people in the Chilean population as of October 2021.^4^ Similar to the situation with SARS in 2002 and a H1N1 influenza in 2009, many healthcare workers have been the front line.^5^

Both pandemics presented a threat for the development of emotional disorders such as stress and anxiety in the healthcare corps from the risk of infecting their families, stigma and post-traumatic stress.^6^,^7^ Healthcare students are a special group to be considered when analysing the consequences of stress and anxiety because of the impacts on learning. A study on nursing students during the H1N1 pandemic determined that the main concerns were infection risk and the negative reactions of other persons that don´t work in health area, evidenced by a behavioural change towards them.^5^ All this fortified students’ professional identity in disciplinary, social, and individual senses, favouring a perception of belonging, connection, morality, and emotionality. By contrast, in the current epidemiological situation, anxiety and fear have been described in nursing students,^8^ being an important substratum for stress, having a great group and individual influence, highlighting the risk of psychological crisis.^8^

During curricular practice, nursing students develop personal confidence and strengthen professional competencies.^9^ However, insecurity, fear, impotence, and uncertainty are important stressors that could worsen clinical experience. There are also high burnout levels,^10^ which is pernicious to professional training.^11^ Reflective learning strategies during the professional education are key for developing professional competence.^12^ Reflective practice stimulate critical examination of self-assumptions, decisions, and conclusions regarding a particular problem, which at the same time, improve clinical judgment by reducing mistakes and increasing capacities. Self-reflection in professional nursing training helps students to develop a greater level of consciousness of events and experiences by expressing feelings and emotions, as well as, validating clinical knowledge, and reflecting on their own behaviour.^13^ In Chile, studies exploring health care student’s perceptions and experiences during this pandemic were not found. Considering the pandemic situation in various hospitals nationwide, there is a need to deepen the meanings in this context for final year nursing students who are providing care to COVID-19 patients. The research question guiding this study is: How is the nursing students’ experience of performing professional experience in the context of COVID-19 Pandemic?

## Methods

Design. A qualitative study design with content analysis^14^,^15^ was conducted based on the self-reflections of fifth-year students performing their professional nursing practice in clinical units where patients with COVID19 were treated. Because of the nature of this study, qualitative methodology is the most appropriate approach for describing and understanding the experiences of the phenomenon under study. The study was guided by the Consolidated Criteria for Reporting Qualitative Research (COREQ). 

Setting and participants. The study was done in a university in Chile, with nursing students, who obtain their degree after 5 years of training. In their last year they complete 540 hours of clinical practice, for 16 weeks. Participant sampling was purposive. All nursing students (*n*=25) doing professional clinical practice in 2 public hospitals and 2 private clinics in Chile, who cared for COVID-19 patients, between the months of March and August 2020 were invited. The study included 13 students, 11 women and 2 men, who voluntarily accepted to participate. Their ages ranged between 22 and 28 years, with average age being 23.4 years. 72% lived with their families, 83% were religious and 2% agnostic, and all were single. The students were invited via email by the secretary of the school of nursing, who is a person external to this study, for diminishing possible conflicts of interest. Subsequently, a videoconference was done to divulge the aim and purpose of this study by the researchers.

Data Collection. Information was gathered through guided written self-reflections by submitting a different open-ended question each week via the Google Forms platform. This was done over a period of 7 weeks until the meaning saturation criterion was reached. Participants were given 500 words to answer the open-ended question. They were invited to find a time of day that best suited them and to create a special space. It was suggested that they light a candle, take a deep breath and inhale to respond from within. The questions that guided students' self-reflections each week were: (1) How has been your experience completing your professional practice during COVID-19 pandemic?, (2) What has meant for you to do your professional practice providing care to COVID patients?, (3) What has been your experience of preserving human dignity during your clinical practice? How have you dealt with it in your personal and professional life?, (4) What and/or who has helped you in difficult moments you have experienced during practice? How has the help been?, (5) How has your learning experience been from the perspective of nursing management, focused specially on patient in Pandemic framework?, (6) How have you worked communication with patients and their families in COVID-19 times?, and (7) What has your experience of writing and self-reflection been like? Written self-reflections provided an opportunity to know students’ thoughts, feelings, emotions, and experiences during their professional practice. It also allowed them to freely express themselves without judgment, as a benefit for the participants. The researchers of the study are female nursing school professors and only they had access to the data collection. All the researchers have clinical and theoretical teaching experience with nursing students. The first two researchers have worked with qualitative methodology before. All of them have a master's degree. Each student was asked to use a pseudonym to protect their anonymity. Reflections were protected with a password and were later erased. The process ended when the meaning saturation criterion was fulfilled^15^ and the researchers collectively decided to finish the reflections. Prior data collection, a pilot test was conducted with two nursing students, were the experience of performing clinical practice with COVID-19 patients was revealed. This information was not included in the analysis.

Data analysis. Qualitative content analysis was done according to the guidelines of Graneheim & Lundman.^14^ Students’ written self-reflections were the analysis unit. The analysis process was inductively and included the following steps: (1) Begin with researcher’s individual reading and re-reading of the reflections one by one, to obtain a global perspective of the contents and familiarize themselves with the information. (2) Meaning units were identified. (3) The condensation process was done, abridging the text, and conserving the essence by making the most faithful possible description of what was said. (4) Next, a code was generated, with the manifest content (the evident) (5) After that, categories were created based on similarities and differences in the content analysis. (6) Finally, the results were discussed between the researchers until they agreed on the codes in discrepancy, categories and themes identified. The analysis was conducted by the investigators. All were nursing professors who supervised the curricular practices. The professors did not know who the participants in the study were, to avoid conflict of interest.

Rigor. Methodological rigour was assured via the criteria of Lincoln and Guba.^15^ *Credibility* was manifested by presenting the results to participants, who recognised themselves in the experience (member checking). The *trustworthiness* criterion was achieved by detailing the methodology and analysis of the study (audit trail). To guarantee confirmability, the entire study process was recorded, giving evidence of participants’ and researchers’ testimony by writing their personal reflections in a field notebook during the study. Finally, to facilitate *transferability* the participants’ characteristics and the study scenario were described in detail. 

Ethical Considerations. This study was approved by the scientific ethics committee of a chilean University. Number of certificates: CEC202043. All study participants previously signed an informed consent release, ensuring their voluntariness, information confidentiality and data anonymisation. 

## Results

Three main themes emerged from the analysis of the written self-reflections describing what this experience meant: (1) facing a highly difficult and stressful situation, (2) having significant support to face difficult moments, (3) learning experience and professional and personal growth. (Table 1).

**Table 1:** Overview of data analysis

Major themes	Subthemes	Codes / unit of meaning
Facing a very difficult and Stressful situation	Global and national Pandemic context: changes or worsens the conditions for clinical practice. Personal protection elements, provide security and tiredness Physical exhaustion, feelings and emotions Ethical-clinical dilemmas around dignity in care and justice	Fear of infecting family. Work shifts. Lack of personal protection elements. Sleepiness. Fear of infection. Uncertainty. Stress Angst. Sorrow, anxiety. Dehumanization around dignity in care. Dying alone. Unequal resource distribution
Recognized the different coping styles in difficult moments.	Contact with significant people. Combination form of support and harmful ways of coping with stress.	Company of the Family, friends, health team, teachers, guiding nurse, patients during process. Phone calls, letters. Words of thanks of the patient. Having faith. Natural medicine and yoga. Consumption of alcohol and sedatives
Experience of disciplinary learning and personal growth	The importance of humanised and transcendent care. Learning from nursing care management. Personal growth during practice Thankfulness and satisfaction for having the opportunity to carry out a clinical internship	Being next to patient. Holding hands. Listening to patient Videocalls. Communication, Interpersonal relations. Action and decision. Leadership. Teamwork. Ethics Time organisation. Care prioritisation. Stress management. Ability to adapt to change. Increased empathy. Understanding and accepting emotions Self-reflection. Being able to accomplish clinical experience Being part of a team. Feeling lucky Feeling happy when caring and treating. Adding my grain of sand

### Theme 1. Facing a very difficult and stressful situation

#### Global and national Pandemic context: changes or worsens the conditions for clinical practice

As the academic year begun in March 2020, the final year nursing students started their professional practice at the same time the COVID 19 pandemic was confirmed worldwide and nationwide. This situation generated changes or worsens the conditions for clinical practice: various hygiene measures with several clothing changes when entering the clinical setting and returning home, changes in the working hours and fear of infecting their families: *Coronavirus cases in Chile and their spread weren’t traceable anymore. That’s where the anxiety started and the fear of entering a clinical setting (E1). Taking the bus and the underground wearing mask and gloves was the weirdest thing I’d done: When I got to the Clinic I changed my uniform and clothes three times a day…when I got home, I did it again... (E2).*

#### Personal protection elements provide security and tiredness

COVID-19 caused the mandatory use of personal protection elements (PPE), posing a challenge for nursing students in their professional practice; extensive workdays led to physical and psychological irritation. However, these measures granted greater security: *My clinical practice become a daily challenge of body, mind and soul…Every day it’s heavier and harder to think…how heavy your legs are, how your hands burn from so much washing, your face hurts from using the mask and the suffocation you get from using it, plus all the aprons and the facial shield (E5).*

#### Physical exhaustion, feelings and emotions

For nursing students, professional practice turned out to be a great challenge involving physical exhaustion and various feelings and emotions: sleeplessness, uncertainty, stress, anxiety, fear, angst, and sadness over their families’ distance: *Physically it’s got really exhausting, since we’ve had to do 24 hour shifts from the first day… talking about the situation with my family generates more chaos, stress, angst and worry (E5); There’s physical and emotional exhaustion within the team… The different protocols and changes we’d face every day made me feel constant angst* (E6); *It’s meant facing my own fears. It was a hard process I never thought I’d go through... (E3).*

#### Ethical-Clinical Dilemma: around dignity in care and justice

Students’ experience involved facing difficult situations related to ethical aspects of patient care, including dehumanisation around dignity in care, facing dying alone, and unequal resource distribution. There were times when they omitted activities that were not a priority, to optimize care times, which was perceived as a form of dehumanization of care, taking distance from the patient, and not delivering comprehensive nursing care: *…I think this pandemic dehumanised care, since it’s so protocolised and you always try to go into the patient’s room the least number of times…forcing you to provide colder and more distant care than we’re used to* (E8); *…I’ve thought about omitting some care, so I can progress with my work and finish on time… (E12).* The dignity of the person was perceived from different perspectives, recognizing its intrinsic value in a particularly vulnerable period. *Dignity is when you make them feel like a person again and you don't treat them like a disease...it is through human interaction that you reach and reinforce that dignity (E6). However, there were times when this was compromised: Patients are treated as mere diseases (E7); Some nurses or technicians are in such a hurry that patient care is not kind or personal (E10).*

For some students was the first encounter with death. It was difficult considering patient’s loneliness and isolation from the loved ones, and not receiving end-of-life care based on human dignity. Some students were irritated and angry because the right of the patient and their family to die with company was violated: *We’ve tried to let the family see them for at least 10 minutes, from a distance with PPE. But I think it’s not enough. How are you going to say goodbye to someone who was important to your life in 10 minutes? That’s the other side of COVID… Then they’re wrapped in the shroud and then in a sort of plastic bag, they’re sealed and then put in the freezer (E9); I feel a deep sorrow in those people who cannot experience a dignified death, being surrounded by a hostile, oppressive, distant and mechanized environment. This is how I would describe the environment that exists in critical patient wards (E4).* Finally, another highly relevant ethical dilemma described by some participants was the way of assigning and using resources among the elderly, generating questions about inequity affecting the principle of justice, where the age of the patient was perceived as an indicator for making decisions: *I’ve felt that doctors try to hurry up some older patients’ deaths… they lower their oxygen to free up the negative pressure room for other patients that’re younger and more critical (E10); Patient’s dignity is violated when we don’t have the means necessary to care for everyone equally… we can’t appropriately care for our population, since we have to choose who we treat and who we won’t (E4).*

### Theme 2. Recognized the different coping styles in difficult moments

#### Significant people 

Students in professional practice had different significant people to better face all the difficult moments previously described. They highlighted support from family, friends, hospital teams, partners going through similar experiences, guiding teachers and instructors: *…Right now I’m getting a lot of support and care from all of my family, friends, teachers, boyfriend, etc. which makes me feel like I’m not alone in this process (E13); I was lucky enough to have an excellent nurse to guide and mentor me who’s supported me unconditionally, as well as a really great teacher, understanding and kind (E7).*

#### Combination form of support and harmful ways of coping with stress

Students described in detail the different forms of support to decrease distress, anxiety and improve sleep, such as: phone calls, letters, patients' words of thanks, the teacher answering her phone after hours, faith, yoga, and natural medicine: My family has been an absolute pillar, in long phone calls, since distance separates us (E3); Patients are very thankful for how we’ve listened to them. That makes me want to carry on, keep going and receiving that affection that is so reassuring, so that, at least for a few hours, all your fears get left aside (E2); The one who’s helped me most in every tough moment, is Jesus… he helps me to find deep meaning at every shift. I personally always have a precious cross in my pocket and every time I’m going through a rough patch, I squeeze it really hard, so I don’t feel alone (E10). They also manifested detrimental ways of coping, behaviour that was subsequently modified: I hit the bottle a lot of times too, one glass as a nightcap to calm the nerves, the anxiety and the angst that would come over me suddenly, or some strong pill to get to sleep. Now I’ve dropped the earlier doses and concentrated on yoga, having more contact with my friends and natural medicine (E5).

### Theme 3. Experience of disciplinary learning and personal growth

#### The importance of humanised and transcendent care

Over time, students caring for patients with COVID19 recognized that care was not only clinical but realized how important it is to provide holistic and humanized care. They identified the emotional and spiritual dimensions through the pain, loneliness, and fear of the patients: *…I’m not talking about putting up a screen, I’m talking about something deeper, interpersonal relations and communication where we can know each other… finding out new needs, giving integral care when I can know the patient, talk about their family, laugh, know their worries, help them. I feel like it was a job well done* (E6). *Entering the room all covered being unable to show a smile, an expression, is tough. Hearing some people saying there’s nothing else to be done, makes me angry and sad…we can always do something else* (E11); *…A patient was really depressed and started to desaturate…she didn’t know how to use the videocalls…I showed her, and she could talk to her kids and grandkids… it changed her mood 100% and her saturation rose* (E10).

#### Learning from nursing care management

For the discipline, learning is described on the base of clinical management, action and decisiveness, leadership, teamwork, learning from the professional and acting ethically with care prioritisation, fortifying, or accelerating the encounter with the essence of care: *It’s hard to organise times and manage patients’ care in an integral fashion… but if the nurse won’t do it nobody will. The spiritual and psychological side strongly influence their recovery* (E10); *For me, doing this clinical experience has meant re-encountering Nursing (E13).*

#### Personal growth during practice

Students on practice referred not only having learned from the perspective of the discipline, but also from a personal growth experience, reflected in adaptation, empathy, flexibility, stress management and frustration: *It’s an experience that taught me a lot, helped me grow and increase my ability to adapt, my empathy and my teamwork* (E6); *It’s true that we’re learning to manage the stress and frustration of situations like what we’re going through, which we probably wouldn’t learn in practice under normal conditions* (E13). Furthermore, students indicated that self-reflection let them put their thoughts in order and express in writing their experiences: *It’s helped me (the self-reflection) to get my head together about what I think and feel, to hear my feelings, to reflect on how I want to be a nurse tomorrow regarding patient treatment…writing reminded me of various scenes that made me explode, which I find good because that way I could express it and release it (E10).*

#### Thankfulness and satisfaction for having the opportunity to carry out a clinical internship 

Finally, in the middle of this adverse context there are thanks, achievements, and satisfaction from students for the opportunity to do their clinical experience reflected in elements such as being part of the team, being on the front line, feeling lucky, being happy giving care, contributing and learning in an unrepeatable context: *At the end of the day I’ve got the chance to make history…I was together with my future colleagues helping my country* (E11): *We all know that what we’re doing is just a drop in the ocean. But if it wasn’t there, the ocean would lack something* (E6).

## Discussion

The objective of this study was to describe the experience of students doing their professional practice in units with patients with COVID-19. Students acknowledged that curricular practice in a pandemic context entailed difficulties and might not have been the most favourable stage for their professional training, coinciding with Peres.^17^ However, for others, it meant personal growth and an encounter with the essence of nursing. A predominant theme in the results of this study coincides with the research conducted in nursing and medical students, who expressed concern about their own health and negative feelings, such as fear of infection, uncertainty and anguish, marking the learning process.^18^,^19^ These same reactions were described by healthcare professionals, who demonstrated increased fear of the risk of accumulating stress-related traumas and psychological complications.^20^ Other aspects included social isolation to avoid contagion, lack of knowledge about this new disease and protocols, and lack of personal protection elements.^21^

Additionally, students’ self-reflections show that they require more tools for deliberating and decision-making regarding complex ethical and clinical situations^22^ and facing dehumanised death processes during the pandemic. 18 A relevant theme that generated doubts among students was the way of assigning and using resources, where patient age is an indicator for taking therapeutic decisions. 23 Another reported result was the need to have significant support to face adverse situations and stress during professional practice. Little information is available regarding how students face clinical practice during COVID-19. However, studies on nurses showed various ways of handling stress and its effects. 24,25

Within the results of this study, faith is described as a form of help in coping with complex moments. This has not been reported in other studies. On the other hand, coinciding with some accounts from the students in this study, a 2021 report on health professionals in Chile revealed an increase in the consumption of alcohol and sedatives during the pandemic.^26^ Therefore, it is necessary to be aware of this reality and identify risk behaviours early to promote healthy lifestyle habits in students. These results suggest that the method of weekly reflections during the clinical practice was very helpful and could be another form of protection and mental health promotion for students.

From the perspective of professional learning, a major challenge in this pandemic is humanising care when facing with the depersonalisation of treatment. This requires nurses who are “partners in human transactions” and can be humanitarian and ethical agents.^27^ From this perspective, students apply knowledge and learn with spiritual and biopsychosocial vision, dedicating quality time and dignified treatment despite the workload and “protocolisation” as the driver of such depersonalisation.

This study has several limitations. This research was done with nursing students, in the intra-hospital setting in Santiago, so generalization could be limited. Students’ experiences in primary care are unknown, as are the experiences of other healthcare students. Most of the participants were women. The researchers were also teachers supervising some of the students on practice, which may have conditioned the students’ self-reflections to be less critical in their discourse. However, their data confidentiality and anonymity were assured.

In conclusion, this study allows us to understand more deeply the students' experience in performing their professional practice during the COVID-19 pandemic. Despite the difficulties, the students recognized the importance of the support received and the provision of humanized care, such as: body language, conscious eye, and physical contact, even without dialogue, establishing itself as an element of relief and a relationship of trust between the patient and the nursing student. The accounts of the students in our study express methods of care oriented to prioritize close treatment, valuing the role of nursing as an integral discipline.

A Brazilian study whose objective was to reflect on the work experienced by nurses in dealing with the COVID-19 pandemic in a public hospital indicated that despite the various barriers: emotional commitment, fear of contagion, lack of human resources and personal protection elements, the nurses were able to practice with satisfaction and leadership, contributing to the improvement of public health.^28^ The experience was an opportunity for personal and professional growth and encountering the essence of nursing. This study also provides relevant information about what could help improve students’ teaching/learning process. Nursing schools should make a strategic plan to support students’ needs and strengthen coping tools in health crisis situations.

Relevance to clinical practice. This study provides relevant information on what could help improve the teaching/learning process for students and thus have a positive impact on the quality of care for patients and their families. Nursing schools should develop a strategic plan to support students' needs and strengthen coping tools in future health crisis situations. The results of this study reinforce the need to address mental health with active self-care strategies for students and health professionals especially in times of crisis.
